# Obituaries

**Published:** 1871-04

**Authors:** 


### OBITUARIES.

KNAPP—Suddenly, on Wednesday, February 15, 1871, Mrs. Emily A., wife of Dr. J. S. Knapp, aged 44 years. Dr. B. M. Palmer officiated at the funeral ceremonies, which took place in the afternoon of the day of her death, at the residence of the family.

### *In Memoriam.*

NEW ORLEANS, Feb. 15, 1871.

At a special meeting of the New Orleans Dental Association, held this day, at the residence of Dr. J. A. DeHart, the following preamble and resolutions were unanimously adopted:

WHEREAS, It has pleased Divine Providence to take from a prominent member of our association, his beloved wife, and we desire to offer our condolence and sympathy to those who are most bereaved. Be it

*Resolved*, That in the sudden and untimely death of Mrs. Jas. S. Knapp, her husband, children, relatives and friends have suffered a deep affliction and an irreparable loss.

*Resolved*, That in the many excellent qualities of mind and heart exhibited by the deceased, are most worthy of emulation, and by her exalted virtues, social and urbane manners, had won the highest esteem of all who knew her.

*Resolved*, That these resolutions be spread upon the minutes of this association, that they be published in the city papers, and that a copy of the same be handed to the family of the deceased.

JOHN G. ANGELL, D. D. S., *President.*

A. F. MCLAIN, D. D. S., M. D., *Rec. Sec.*

---

DIED—On the morning of February 14, after a short illness, our highly esteemed and worthy professional brother, Dr. H. E. Peebles, of St. Louis, Mo.

We have known Dr. Peebles for many years, as a most noble Christian gentleman. He was appreciated and loved wherever known; and he was well known to the dental profession, not only of this country but in other countries. In relation to his death the following action was had at the late meeting of the Ohio Dental College Association: T.

*Resolved*, That in the death of Dr. H. E. Peebles, the association has lost one of its most active and useful members; the institution one of its first and warmest patrons, Dr. Peebles being one of its first stockholders; and the profession at large, a high minded and honorable member. And as a memorial of our estimation of his worth, we place this resolution on our minutes, and that our Corresponding Secretary send a copy to his family.

JAMES TAYLOR.
JAMES LESLIE.

